# Mr. Harrup on the Electrical Machine

**Published:** 1812-10

**Authors:** Robert Harrup

**Affiliations:** Chobham


*To the Editors of the Medical and Physical Journal.*

GENTLEMEN,

YOUR Correspondent, Mr. Phythian, in your last Number, is anxious for an answer to a question in your twenty-sixth Volume, which, at page 110, I find to be,—

 What

What is the most advantageous form for an electrical machine, and what are the reasons for such a peculiarity of construction?

I apprehend that any well made machine, whatever may be its construction, if kept in proper order, will answer all the purposes of electrization. But there are other circumstances to be attended to, both for the convenience of the operator and for the ease of the patient.

The cylinder machine is that which is most commonly used in this country. In it, however, we frequently find much want of uniformity in the supply of the electric fluid, either from the irregularity of the cylinder, or from the unequal pressure of the cushion, so that a portion is dissipated in the air or on other surrounding bodies. Where no regard is had to expense, this defect may, indeed, be remedied, but I allude to those which are generally to be found in the shops.

Other objections may be made to this form when employed for medical purposes, but that which appears to be of the most consequence is the bulk of the whole apparatus when packed in the box. Many attempts have been made to reduce cylinder machines to a portable size, but, I believe, hitherto without effect. It seems impossible to reduce such to a convenient size, and to be at the same time of any use.

The plate-machine, as improved by Van Marum, is in all respects preferable to every other for the use of medical practitioners. It is superior in steadiness of action and intensity to any cylinder machine, and far exceeds in the power of charging. In order to shew the superiority in charging by the improved plate machine, Van Marum made a comparative trial between his and the great Teylerian plate machine, in its original state, before his improved rubbers were applied to it. His plate was thirty-one inches diameter, and, although the experiment was made in very unfavorable circumstances, he found that 150 turns of the plate charged a battery of ninety jars, each containing upwards of a square foot of coated glass, to the highest degree, so that it discharged itself. The great Teylerian machine, with two plates of sixty-five inches diameter, never charged the same battery, in the most favorable circumstances, in less than sixty-six turns, consequently this small machine exhibited about $\frac{4}{9}$ths of the power of the great one in its unimproved state. Had all the circumstances been alike favorable, the power displayed would, probably, have amounted to one half.

Many

Many years ago a plate machine was constructed by a person of the name of Swift, which packed into a box of the form of two folio volumes, lettered on the backs—*Swift's Works.* As this was several years prior to Van Marum's improvement, I suppose it was liable to all the objections of such machines at that time, consequently never came into general use.

Perhaps an ingenious workman might find his advantage in constructing an improved plate machine to pack into a box, similar to that of Swift. The medical apparatus, not being extensive, would occupy but a small space. The plate might be of a moderate size and yet answer every intention, and the medical jar, &c., which is now cylindrical, consequently occupying more space, might be made elliptical without diminishing its properties.

With respect to the insulating stool, those which generally accompany the machines in common use are much too small, being only barely of a size for a person to stand upon. It is well known to every practitioner that in a great number of cases in which electrization is employed, the patient is unable to stand at all, much less on a small stool, and that very few are able to continue standing during the time which may be necessary for insulation.

For many years past I have used an insulating stool of such dimensions as to place a chair upon it, but, as any thing of this kind is inadmissible where portability is the first consideration, I would propose, as a substitute, six circular pieces of well varnished glass, about two inches in depth and three over. A chair to be placed upon four of these, and two for the patient to rest the feet upon. Having formerly used such, I can affirm from experience that, insulation may be obtained by them equal to the stool generally used. If, when in use, a piece of very dry writing paper, a few inches square, be placed under each, the insulation will be more complete. I presume that an electrical machine and apparatus for medical purposes *alone,* constructed on these principles, would be sufficiently portable and answer every intention, as well, if not better, than the largest.

Considering the improved state of science, it is surprizing that so little attention has been bestowed on electricity as a remedy. The extraordinary cures which at different times it has effected ought to have been a sufficient inducement to practitioners to give it a fair trial in all obstinate cases where there were the least prospect of success. The general complaint is, that it is not uniform in its good effects;

 but, but, if this is to be admitted as an objection, many other powerful remedies would have been long since laid aside. The truth is, that formerly much more was expected from electricity than could possibly be effected by it or any other remedy, and the consequence was, it fell into disrepute.

Our present knowledge of this remedy is extremely limited, to ascertain its powers in this respect we ought to begin, as it were, *de novo*, and whatever its effects may be, they should be given to the public. One fact is certain, that it may be brought to act on every part of the body, however remote from the surface, and probably, by its continued application, diseased action may be either changed, or, at least, suspended. During twenty-five years practice I have employed electricity in a variety of cases, as opportunity offered, and the general result of my experience is, that it is well deserving the attention of every medical practitioner, and that it will sometimes effect more than any other known remedy. Before concluding, I take the liberty of requesting the attention of the profession to a fact very little noticed, and, perhaps, not generally known. I allude to that of administering mercury, where nothing contra-indicates, during a course of electrization. If mercury is contained in the system, electrical shocks or sparks produce a much more disagreeable and painful sensation than in other instances. From the case mentioned by Mr. Hunter, in which electrization had no effect till mercury was administered, and which afterwards produced a cure, I was, many years ago, led to try this method, and am persuaded that the employment of the mercurial during the electrical course is attended with considerable advantage indeed. From the trials I have made, it appears, that more may be effected by the employment of both at the same time, than by either of the remedies used separately. Aware, however, of the vast power of mercury *alone* in removing diseased action, I wish not to be understood as speaking with certainty on this fact.

The identity of galvanism and electricity is now pretty well ascertained, what may be the effect of this modification of the latter in curing diseases, future experience can alone determine; however that may turn out, it is not probable that galvanism will come into general use in the present mode of exhibiting it.

I am, Gentlemen,

Your most obedient Servant,

ROBERT HARRUP.

*Chobham*, *June* 17, 1812.

